# Chemotherapeutic Drug Resistance Associated with Differential miRNA Expression of miR-375 and miR-27 among Oral Cancer Cell Lines

**DOI:** 10.3390/ijms24021244

**Published:** 2023-01-08

**Authors:** Kieran Caberto Huni, Jacky Cheung, Madeline Sullivan, William Taylor Robison, Katherine M. Howard, Karl Kingsley

**Affiliations:** 1 Department of Advanced Education in Orthodontic Dentistry, School of Dental Medicine, University of Nevada-Las Vegas, 1700 W. Charleston Boulevard, Las Vegas, NV 89106, USA; 2 Department of Clinical Sciences, School of Dental Medicine, University of Nevada-Las Vegas, 1700 W. Charleston Boulevard, Las Vegas, NV 89106, USA; 3 Department of Biomedical Sciences, School of Dental Medicine, University of Nevada-Las Vegas, 1001 Shadow Lane, Las Vegas, NV 89106, USA

**Keywords:** oral squamous cell carcinoma, molecular biology, biomarkers, chemoresistance

## Abstract

Recent advances have suggested that non-coding miRNAs (such as miR-21, miR-27, miR-145, miR-155, miR-365, miR-375 and miR-494) may be involved in multiple aspects of oral cancer chemotherapeutic responsiveness. This study evaluated whether these specific miRNAs are correlated with oral cancer responsiveness to chemotherapies, including Paclitaxel, Cisplatin and Fluorouracil (5FU). Commercially available and well-characterized oral squamous cell carcinoma cell lines (SCC4, SCC9, SCC15, SCC25 and CAL27) revealed differing resistance and chemosensitivity to these agents—with SCC9 and SCC25 demonstrating the most resistance to all chemotherapeutic agents. SCC9 and SCC25 were also the only cell lines that expressed miR-375, and were the only cell lines that did not express miR-27. In addition, the expression of miR-375 was associated with the upregulation of Rearranged L-myc fusion (RLF) and the downregulation of Centriolar protein B (POC1), whereas lack of miR-27 expression was associated with Nucleophosmin 1 (NPM1) expression. These data have revealed important regulatory pathways and mechanisms associated with oral cancer proliferation and resistance that must be explored in future studies of potential therapeutic interventions.

## 1. Introduction

Increasing evidence has demonstrated that resistance to chemotherapy remains one of the most important challenges facing oral cancer treatment and oncology [1,2]. These observations have propelled efforts to elucidate the mechanisms that may be responsible for oral cancer chemotherapeutic resistance [3,4]. Although multiple mechanisms have been identified, many studies have now identified miRNAs as critical intermediaries that function to modulate many of the pathways closely associated with chemotherapeutic resistance among oral cancers [5,6,7].

Many reviews of miRNA expression among oral cancers have identified the potential utility of miRNA expression profiles as biomarkers and determinants for chemotherapy treatment [8,9]. This has led to the identification of specific miRNAs that modulate multiple tumorigenesis pathways, as well as resistance to chemotherapeutic agents—such as miR-21 and chemoresistance to Cisplatin therapy [10,11,12]. An additional well-established miRNA that modulates tumorigenesis, progression and chemoresistance is miR-365, which mediates differentiation, proliferation and survival among oral cancers—as well as resistance to Fluorouracil (5FU) therapy [13,14,15].

Although progress has been made towards the identification of common miRNA expression profiles that influence oral cancer growth, metastasis and chemotherapeutic resistance, recent advances have suggested that additional non-coding miRNAs (such as miR-155 and miR-494) may be involved in multiple pathways more specifically related to chemotherapeutic responsiveness to Paclitaxel [16,17]. In addition, new research has revealed other miRNAs that may modulate chemotherapy resistance in oral cancers, such as miR-27 and Cisplatin resistance [18,19]. Other studies have demonstrated potential associations between oropharyngeal and esophageal cancer chemotherapy and radiation resistance with miR-125, miR-143 and miR-375 [20,21,22]. 

In fact, reviews of large-scale studies have revealed that the potential list of miRNAs that may functionally participate in chemotherapy resistance has grown significantly over the past few years [23,24]. Although some studies provide evidence of oral cancer chemotherapy resistance associated with miRNA expression, there remains a lack of evidence that examines the potential pathways and mechanisms responsible for these observations [25,26,27,28]. Based upon the paucity of information regarding this phenomenon, the overall goal of this project is to determine whether specific miRNAs are correlated with oral cancer responsiveness to chemotherapies among well-characterized oral cancer cell lines, including Paclitaxel, Cisplatin and Fluorouracil (5FU)—as well as an examination of the potential pathways and mechanisms that may be responsible for these observations.

## 2. Results

**Figure 1:**
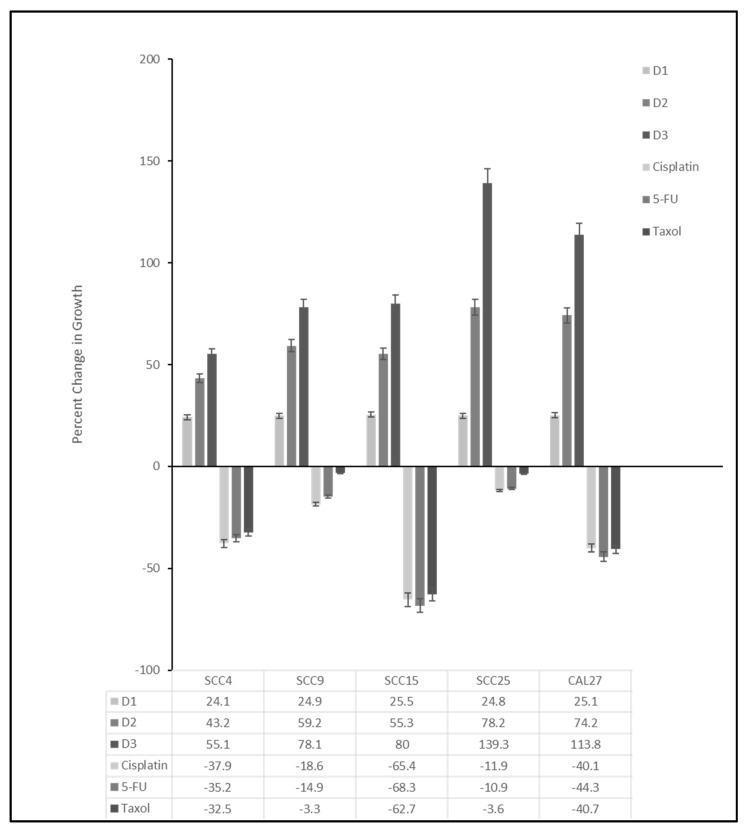
Growth rates and chemotherapy resistance of oral cancer cell lines. Three-day proliferation assays revealed rapid growth with the highest percent change among SCC25 (139.3%) and CAL27 (113.8%) cells. Administration of chemotherapy agents Cisplatin, 5-FU (Fluorouracil) and Taxol (Paclitaxel) demonstrated robust inhibition among SCC15 cells (−65.4%, −68.3% and −62.7%) but not SCC9 (−18.6%, −14.9% and −3.3% *) or SCC25 cells (−11.9%, −10.9% and −3.6% *), which were all statistically significant except those denoted by * (*p* > 0.05).

Cell cultures of all oral cancers were established and proliferation rates determined using 96-well three-day growth assays (Figure 1). These data demonstrated that SCC4 exhibited the slowest growth from an increase of 24.1% on Day 1 (D1) to 55.1% on Day 3 (D3). The most rapid growth was observed with SCC25 cells with an increase of 24.8% on D1 to 139.3% on D3 and CAL27 cells with increases of 25.1% on D1 and 113.8% on D3.

To determine whether these cell lines exhibited chemotherapy resistance, cells were plated in three independent experiments with and without Cisplatin, 5-FU (Fluorouracil) and Taxol (Paclitaxel). These data demonstrated that the most inhibition (percent change between control and experimental conditions) was observed with SCC15 cells, with decreased growth of 65.4% (*p* = 0.0012), 62.7% (*p* = 0.0014) and 68.3% (*p* = 0.0011). In contrast, two cell lines exhibited the least inhibition (most chemotherapy resistance to Cisplatin, 5-FU and Taxol), including SCC9 (18.6%, *p* = 0.018; 14.9%, *p* = 0.022; and 3.3%, *p* = 0.065, respectively) and SCC25 (11.9%, *p* = 0.039; 10.9%, *p* = 0.042; and 3.6%, *p* = 0.061, respectively).

To evaluate if miRNA expression differs among the oral cancer cell lines, RNA was extracted from each cell culture (Table 1). These data revealed an average RNA concentration of 501.6 ng/uL that ranged between 488.1 and 515.3 ng/uL, which was well within the expected concentration range of the manufacturer protocol specifications (100–1000 ng/uL). The purity of RNA was evaluated using the ratio of absorbance at A260 nm and A280 nm, which revealed an average ratio of 1.77 that ranged between 1.71 and 1.83. Additionally, screening at A230 nm to evaluate the amount of organic (phenol) carryover revealed minimal contamination, with an average A260:A230 ratio of 2.08 that ranged from 2.02 to 2.14.

The RNA isolated from each cell line was converted into cDNA and amplified to enable qPCR screening of low-expression miRNA targets (Table 2). These data revealed successful cDNA and synthesis reactions from all cell lines, averaging 1496.6 ng/uL that ranged between 1438.4 and 1566.2 ng/uL. DNA purity was calculated using A260:A280 ratios, which averaged 1.84 and ranged between 1.81 and 1.88.

**Figure 2:**
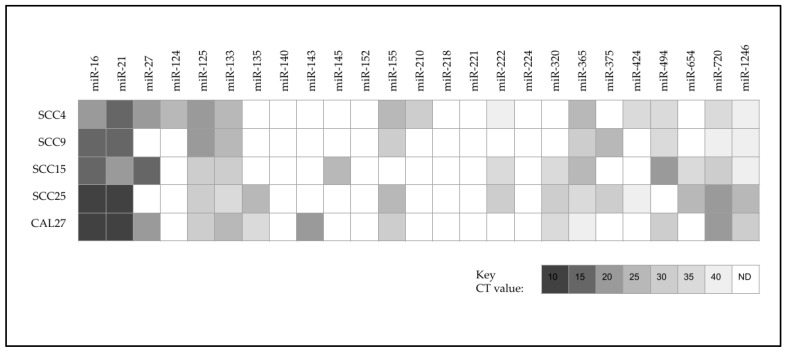
qPCR screening for miRNA expression among oral cancer cell lines. Screening of cDNA revealed expression of miR-16, miR-21, miR-125, miR-133, miR-365, miR-720 and miR-1246 among all cell lines. No expression of miR-140, miR-152, miR-218, miR-221 and miR-224 was detected in any of the cell lines evaluated. Differential expression was observed with miR-27, miR-124, miR-135, miR-143, miR-145, miR-155, miR-210, miR-222, miR-320, miR-375, miR-424, miR-494 and miR-654. N.D. = not detected.

Screening of cDNA using validated qPCR primers for miRNA expression was performed (Figure 2). These data revealed the expression of the miRNA positive control miR-16 among all cell lines. In addition, several miRNAs were expressed among all cell lines, including miR-21, miR-125, miR-133, miR-365, miR-720 and miR-1246—although the differential expression was observed. Several miRNAs were not expressed among any of the cell lines evaluated, including miR-140, miR-152, miR-218, miR-221 and miR-224.

Differential expression was observed with miR-27, miR-124, miR-135, miR-143, miR-145, miR-155, miR-210, miR-222, miR-320, miR-375, miR-424, miR-494 and miR-654. More specifically, expression of miR-143 was found only with CAL27, miR-145 was found to be expressed only in SCC15, and both miR-124 and miR-210 were only expressed in SCC4. miRNAs that were only present in two cell lines included miR-375 (SCC9 and SCC25), miR-424 (SCC4 and SCC25) and miR-654 (SCC15 and SCC25), while miRNAs expressed in three cell lines included miR-27 (SCC4, SCC15 and CAL27), miR-135 (SCC4, SCC25 and CAL27), miR-222 (SCC4, SCC15 and SCC25) and miR-320 (SCC15, SCC25 and CAL27). Finally, miRNAs expressed in four cell lines included miR-155 (expressed in all but SCC15) and miR-494 (expressed in all but SCC25).

**Figure 3:**
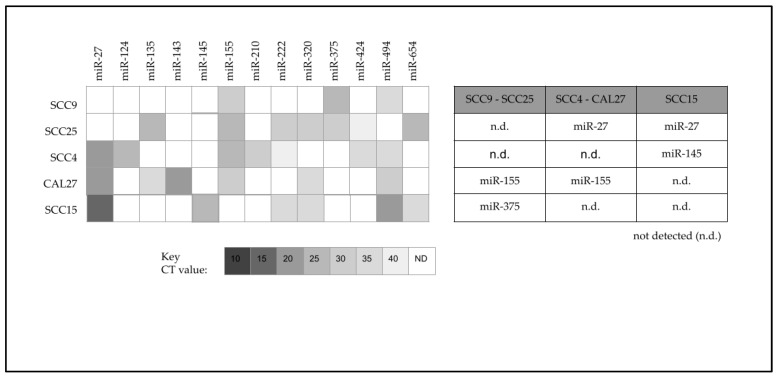
Differential expression of miRNA among oral cancer cell lines. Analysis of the most chemoresistant cell lines (SCC9 and SCC25) revealed differential expression of miR-375 with no expression of miR-27. The least resistant cell line (SCC15) exhibited differential expression of miR-145 with no expression of miR-155.

To more effectively evaluate the relationship between chemotherapy resistance and miRNA expression, the differentially expressed miRNA data were graphed against the most resistant (SCC9 and SCC25), moderately resistant (SCC4 and CAL27) and least resistant (SCC15) cell lines (Figure 3). These data revealed distinct expression patterns associated with the most resistant cell lines SCC9 and SCC25, which were the only cell lines that did not express miR-27. In addition, these cell lines (SCC9 and SCC25) were also the only lines to express miR-375.

Although these expression patterns among the most resistant cell lines were found, an additional pattern was found among the least resistant cell line SCC15. More specifically, SCC15 was the only cell line to express miR-145. Finally, SCC15 was the only cell line not found to express miR-155, in stark contrast with the other cell lines.

**Figure 4:**
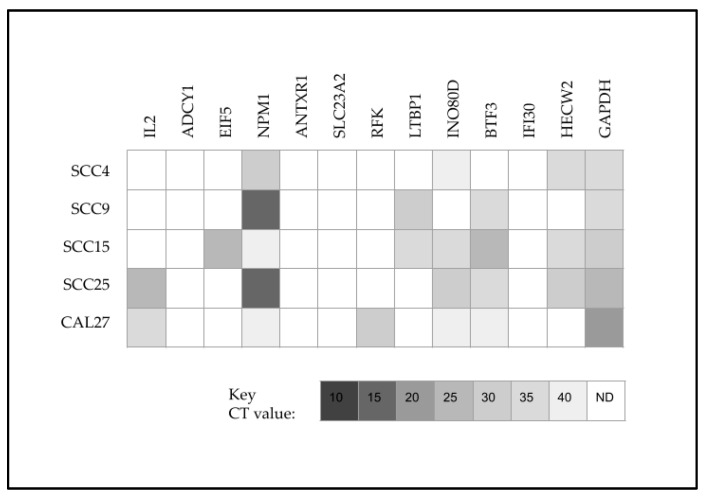
Screening of predicted downstream targets for miR-27. All cell lines expressed the positive control GAPDH and NPM1 with highest expression found in SCC9 and SCC25 cells. Expression of EIF5 was found among SCC15 cells with RFK only observed with CAL27 cells. None of the cell lines evaluated expressed ADCY1, ANTXR1, SLC23A2 or IFI30.

To assess the effects of these differential miRNA expression patterns, the predicted downstream targets for miR-27 (with target scores > 90) were evaluated and screened using qPCR (Figure 4). These data demonstrated that none of the cell lines evaluated expressed Adenylate cyclase 1 (ADCY1), Anthrax Toxin Receptor cell adhesion molecule 1 (ANTXR1), Solute carrier family 23 member 2 (SLC23A2) or Lysosomal thiol reductase (IFI30). In contrast, all cell lines were found to express Nucleophosmin 1 (NPM1)—although differential expression was observed, with the most robust expression found among SCC9 and SCC25 cells. Expression of Eukaryotic translation initiation factor 5 (EIF5) was found only among SCC15 cells, while expression of Riboflavin kinase (RFK) was found only among CAL27 cells. 

**Figure 5:**
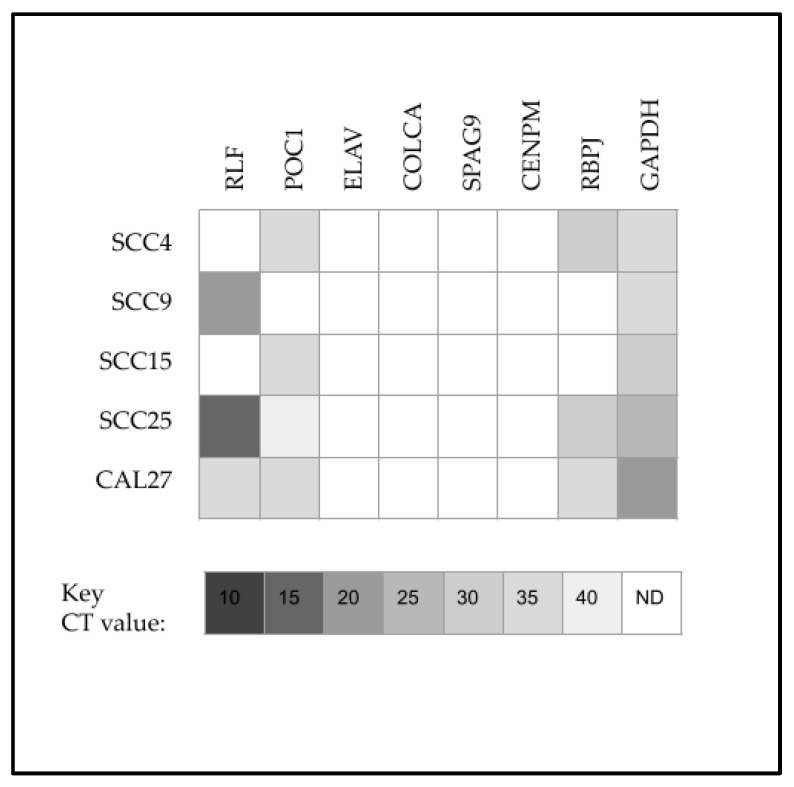
Screening of predicted downstream targets for miR-375. All cell lines expressed the positive control GAPDH cells with none of the cell lines expressing ELAV, COLCA, SPAG9 or CENPM. Differential expression was observed with RLF (SCC9 and SCC25 highly expressing), POC1 (SCC9 and SCC25 not expressing) and RBPJ.

To assess the effects of these differential miRNA expression patterns, the predicted downstream targets for miR-375 (with target scores > 90) were evaluated and screened using qPCR (Figure 5). These data demonstrated that none of the cell lines evaluated expressed Embryonic lethal abnormal vision-like RNA binding protein 4 (ELAV), Colorectal cancer associated 2 (COLCA), Sperm associated antigen 9 (SPAG9), or Centromere protein M (CENPM). In contrast, differential expression was observed with Rearranged L-myc fusion (RLF), Centriolar protein B (POC1) and Recombination signal binding protein (RBPJ). More specifically, the expression of RLF was more robust among SCC9 and SCC25 cells, with lower expression observed among CAL27 and no expression among SCC4 or SCC15 cells. In addition, POC1 was not expressed among either SCC9 or SCC25 cells, but was found among all the other oral cancer cell lines.

To more directly assess the potential association between miRNA expression and chemotherapy resistance, the key results of miR-27 and miR-375 downstream targets were assembled (Table 3). These data demonstrated that the expression of miR-27 may be negatively associated with the expression of Nucleophosmin 1 (NPM1) in both SCC9 and SCC25, but not SCC4, CAL27 or SCC15 cells, which have a significant negative Pearson correlation with average chemotherapy resistance, R = −0.9789. In addition, the expression of miR-375 may be positively associated with the expression of Rearranged L-myc fusion (RLF) in both SCC9 and SCC25, but not SCC4, CAL27 or SCC15 cells, which have a significant positive correlation with average chemotherapy resistance, R = 0.9583. However, expression of miR-375 may be negatively associated with Centriolar protein B (POC1) expression in SCC9 and SCC25, but not SCC4, CAL27 or SCC15 cells, which have a significant negative correlation with average chemotherapy resistance, R = 0.9754.

## 3. Discussion

Due to the expanding evidence that miRNAs are associated with chemotherapy resistance, the overall goal of this project was to evaluate both oral cancer resistance and miRNA expression to determine whether significant relationships could be identified. The results of this study clearly demonstrated at least two oral cancer cell lines (SCC9 and SCC25) with significant resistance to all three main chemotherapies (Cisplatin, 5-FU and Taxol), confirming recent observations made in other studies of chemotherapy resistance [29,30,31,32]. These data contribute to a body of important research that seeks to identify model systems for evaluating the mechanisms responsible for chemotherapy resistance among oral cancers [33].

The evaluation of miRNA expression among these chemoresistant cell lines demonstrated a positive association with miR-375 expression, which may be one of the first studies to validate this result in a commercially available cell line [20,21,22]. Studies of miR-375 among liver cancers have demonstrated that this resistance may function through modulation of Non-SMC Condensin II Complex Subunit G2 (NCAPG2), Interleukin 6 (IL-6) and Transforming growth factor-beta (TGF-β) [34,35]. Additional research studies of osteosarcoma have demonstrated that miR-375 may function through interactions with Autophagy related gene 2B (ATG2B) and Myeloid leukemia cell differentiation protein (Mcl-1) [36,37]. Finally, studies of prostate and colorectal cancers have demonstrated that miR-375 may participate in chemoresistance through interactions with Yes-associated protein 1 (YAP1) and Specificity Protein 1 (SP1) transcription factors [38,39].

This study may be the first to demonstrate a positive association between miR-375 and Rearranged L-myc fusion (RLF) transcription factor expression in chemoresistant oral cancers, although one previous study of pig Sertoli cells found a similar association between miR-375, proliferation rates and RLF expression [40]. In addition, a previous screening of melanomas demonstrated differential RLF expression, although no mechanism of RLF expression, such as miRNA regulation, was identified [41]. As RLF has been identified as a potent effector of gene expression and cell growth via the well-characterized Rat sarcoma (RAS), mitogen activated protein kinase (MAPK) and phosphoinositide-3 kinase (PI3K)-specific growth and survival pathways, these results suggest that miR-375 and RLF expression may therefore represent part of the functional relationship between oral cancer growth, proliferation and chemotherapy resistance [42].

This study also found an inverse relationship between miR-375 and Centriolar protein B (POC1) expression. POC1 has been identified as a protein mediator of centriole formation and stability, an important functional role in rapidly dividing cells, including cancer cells [43]. However, other studies have also found overlapping functions between additional POC-family member proteins with compensatory mechanisms that may serve as functional centriole- and centrosome-organizing alternatives or substitutes should any one POC family member be dysfunctional or dysregulated, such as POC1 [44]. 

Finally, these results also demonstrated that miR-27 expression was associated with NPM1 expression. Nucleophosmin 1 (NPM1), also known as nucleolar phosphoprotein B23 or numatrin, has been implicated in the development of cancer [45,46]. More specifically, NPM1 has multiple functions involving nucleolus organization and function and has been identified as overexpressed in clinical studies of gene dysregulation among oral cancers [47,48]. This study may be the first to identify the association between NPM1 expression and the lack of miR-27 expression, which may help with the identification of molecular mechanisms that target NPM1 expression and might ultimately lead to the development of specific functional therapies [49].

Although these represent significant findings, this study had several limitations that should also be considered. For example, in order to establish a model system for studying these effects, this study involved the use of commercially available oral cancer cell lines, as has been done in other studies evaluating chemotherapeutic resistance [50]. While this may further expand the potential list of biomarkers available for oral cancers, these results must be subsequently validated among primary tumors and explants [51]. In addition, the association between miRNA expression (or lack of expression) and these functional targets must be investigated in subsequent studies to determine if overexpression or underexpression demonstrate similar functional relationships [52,53].

## 4. Materials and Methods

### 4.1. Experimental Cell Lines

Cell lines used in this study were commercially available, well-characterized oral squamous cell carcinoma (OSCC) lines, including SCC-4 (CRL-1624), SCC-9 (CRL-1629), SCC-15 (CRL-1623), SCC-25 (CRL-1628) and CAL27 (CRL-2095) obtained from American Culture Tissue Collection (ATCC; Manassas, VA, USA). Cells were maintained according to the manufacturer recommendations, which included Dulbecco’s Modified Eagle’s Medium (DMEM) for CAL27 cells and DMEM:F12 for SCC-4, SCC-9, SCC-15 and SCC-25 cells, supplemented with 10% fetal bovine serum (FBS) and 1% penicillin-streptomycin—all obtained from ThermoFisher Scientific (Fair Lawn, NJ, USA). All cell cultures were maintained in 25 cm2 tissue-culture treated flasks in a humidified BiosafetyLevel 2 (BSL-2) incubator at 37 °C with CO2 supplemented at 5%. Verification of each cell line was provided by the manufacturer using the Short Tandem Repeat (STR) method to provide validity (>90%) for each cell line prior to use, as previously described [14,15,27].

STR cell line validation and cross check from manufacturer:

**SCC-4 (CRL-1624)****SCC-9** **(CRL-1629)****SCC-15 (CRL-1623)****SCC-25** **(CRL-1628)****CAL-27** **(CRL-2095)**STR profile analysisAmelogenin: X, Y CSF1PO: 11 D13S317: 11,13 D16S539: 12 D5S818: 13 D7S820: 9,11 THO1: 9.3 TPOX: 8 vWA: 15,17Amelogenin: X, Y CSF1PO: 11 D13S317: 9 D16S539: 10,11 D5S818: 12 D7S820: 8 THO1: 8,9 TPOX: 9,11 vWA: 17Amelogenin: X, Y CSF1PO: 10,13 D13S317: 9,14 D16S539: 12,15 D5S818: 12 D7S820: 10,11 THO1: 9,9.3 TPOX: 8 vWA: 15,17Amelogenin: X CSF1PO: 10 D13S317: 13 D16S539: 11,12 D5S818: 12 D7S820: 12 THO1: 8 TPOX: 8,12 vWA: 17,19Amelogenin: X CSF1PO: 10,12 D13S317: 10,11 D16S539: 11,12 D5S818: 11,12 D7S820: 10 THO1: 6,9.3 TPOX: 8 vWA: 14,17STR% match92%100%94%100%93%Cell typeOral squamous cell carcinomaOral squamous cell carcinomaOral squamous cell carcinomaOral squamous cell carcinomaOral squamous cell carcinoma

### 4.2. Experimental Agents

Proliferation assays were performed using experimental anti-tumor chemotherapeutic agents, including Cisplatin or cis-diammine-dichloro-platinum (NSC 119875; MW 300.5) from Selleck Chemical (Houston, TX, USA), 5-FU or 5-Fluorouracil 5-FU (NSC 19893; MW 130.08) and Paclitaxel or Taxol (NSC 125973; MW 853.91). Concentrations evaluated ranged between 1.0 ng/mL (low), 5.0 ng/mL (mid) and 10.0 ng/mL (high), which were used to approximate the physiologic doses and bioavailability studies [27,28].

### 4.3. Proliferation Assays

Experimental 96-well assays were performed to determine chemotherapy resistance and sensitivity. In brief, each oral cancer cell line was plated with and without chemotherapy anti-tumor agents Cisplatin, 5-Fluorouracil or Paclitaxel at three concentrations over the range of 1.0 (low), 5.0 (mid) and 10.0 (high) ng/mL. Cells were plated using *n* = 8 replicates at standard concentrations of 1.2 × 10^4^ cells/mL with and without these chemotherapeutic agents for 24 h (1 day), 48 h (2 days) and 72 h (3 days). Three separate, independent experiments were conducted for each cell line at each concentration using each chemotherapeutic agent. At the conclusion of each assay, cells were fixed with 10% buffered formalin for 24 h prior to staining with Gentian Violet 1% aqueous solution from Ricca Chemical (Arlington, TX, USA). Plates were processed using an ELx808 Absorbance Microplate Reader from BioTek (Winooski, VT, USA) at 630 nm, as previously described [28.29].

### 4.4. RNA Isolation

Total cellular RNA was isolated from each experimental cell line for further analysis. The phenol:chloroform extraction method was used with the TRIzol reagent from Invitrogen (Waltham, MA, USA). Cellular lysates were transferred to sterile microcentrifuge tubes (1.0 mL) with 200 uL of chloroform added. Samples were mixed and stored on ice for 15 min prior to centrifugation at 12,000× g or relative centrifugal force (RCF) using the 5424-R Refrigerated Microcentrifuge from Eppendorf (Hamburg, Germany). The upper RNA-containing aqueous phase was transferred to a new sterile microcentrifuge tube with an equal volume of isopropanol to precipitate the nucleic acids prior to centrifugation for 10 min at 4 °C. The isopropanol was removed and the pellet washed with ethanol prior to a final centrifugation for five minutes at 4 °C. The ethanol was removed and the pellet was resuspended in nuclease-free distilled water. 

Concentration of RNA was evaluated using the NanoDrop 2000 Spectrophotometer from Fisher Scientific (Fair Lawn, NJ, USA). Absorbances at A260 nm and A280 nm were assessed to determine the relative abundance or concentration of RNA, as well as the overall quality of each sample. RNA samples with sufficient concentration (>100 ng) and A260:A280 ratios above 1.65 were deemed sufficient for subsequent analysis.

### 4.5. cDNA Synthesis and qPCR Screening

All samples of RNA isolated from each cell line were reverse transcribed using the Verso 1-step RT-PCR kit from ThermoFisher (Fair Lawn, NJ, USA). In brief, the cDNA synthesis was completed using a Mastercycler thermal cycler from Eppendorf (Hamburg, Germany) using 25 uL of 1-Step ReddyMix, 1.0 uL of Verso Enzyme Mix, 2.5 uL of RT enhancer, 1.0 uL of Universal Forward and Reverse Primers, 1.0 uL of RNA template, and 20 uL of nuclease-free water. Synthesis settings included 15 min of cDNA synthesis at 50 °C and 2 min of enzyme deactivation at 95 °C, followed by 40 cycles including 20 s of denaturation at 95 °C, 30 s of annealing at primer pair-specific temperature and 60 s of extension at 72 °C.

To enable the amplification of low expression miRNA targets, cDNA from the amplification reaction was processed using the TaqMan miR-Amp Reaction Mix from Applied Biosystems (Waltham, MA, USA). Briefly, miR-Amp Master Mix (2X), Primer Mix (20X) and RNase-free water were prepared with cDNA from the cDNA synthesis reaction and amplified using a thermal cycler and one cycle of 95 °C for five minutes for enzyme activation, and 14 cycles of denaturation at 95 °C with annealing and extension at 60 °C for 30 s, followed by a stop reaction at 99 °C for ten minutes.

The QuantStudio Real-Time Polymerase Chain Reaction (PCR) system from Applied Biosciences (Waltham, MA, USA) was used to facilitate sample screening. The reactions for qPCR reactions used the SYBR Green qPCR Master Mix from ThermoFisher Scientific (Fair Lawn, NJ, USA). Each reaction contained 12.5 uL of Absolute SYBR Green, 1.75 uL each of forward and reverse primers, 7.5 uL of nuclease-free water and 1.5 uL sample DNA to complete a 25 uL reaction volume. Specifications for thermocycling involved enzyme activation for 15 min at 95 °C, with 40 cycles involving denaturation for 15 s at 95 °C, annealing for 30 s using each primer pair-specific temperature, and final extension for 30 s at 72 °C. Validated primer sets included [27,28]:

GAPDH (metabolic) control primers
GAPDH forward: 5′ATCTTCCAGGAGCGAGATCC-3′; 20 nt, 55% GC, Tm: 66 °C
GAPDH reverse: 5′ACCACTGACACGTTGGCAGT-3′; 20 nt, 55% GC, Tm: 70 °C
Beta-actin (structural) control primers
Beta-actin forward: 5′-GTGGGGTCCTGTGGTGTG-3′; 18 nt, 67% GC, Tm: 69 °C
Beta-actin reverse: 5′-GAAGGGGACAGGCAGTGA-3′, 18 nt, 61% GC, Tm: 67 °C

miR-16
miR-16 forward: 5′-TAGCAGCACGTAAATATTGGCG-3′; 22 nt, 45% GC, Tm: 65 °C
miR-16 reverse: 5′-TGCGTGTCGTGGAGTC-3′; 16 nt, 63% GC, Tm: 65 °C

miR-21
miR-21 forward: 5′-GCCACCACACCAGCTAATTT-3′; 20 nt, 50% GC, Tm: 66 °C
miR-21 reverse: 5′-CTGAAGTCGCCATGCAGATA-3′; 20 nt, 50% GC, Tm: 65 °C

miR-27
miR-27 forward: 5′-ATATGAGAAAAGAGCTTCCCTGTG-3′; 24 nt, 42% GC, Tm: 61 °C
miR-27 reverse: 5′-CAAGGCCAGAGGAGGTGAG-’3′; 18 nt, 61% GC, Tm: 67 °C

miR-124
miR-124 forward: 5′-TTCACAGCGGACCTTGA-3′; 17 nt, 53% GC, Tm: 64 °C
miR-124 reverse: 5′-GAACATGTCTGCGTATCTC-3′; 19 nt, 47% GC, Tm: 60 °C

miR-125
miR-125 forward: 5′-GCCCTCCCTGAGACCTCAA-3′; 19 nt, 63% GC, Tm: 69 °C
miR-125 reverse: 5′-GTGCAGGGTCCGAGGT-3′; 16 nt, 69% GC, Tm: 68 °C

miR-133
miR-133 forward: 5′-CCGGTTAACTCGAGCTCTGTGAGAG-3′; 25 nt, 56% GC, Tm: 71 °C
miR-133 reverse: 5′-CTAGCTAGGAATTCTGTGACCTGTG-’3′; 25 nt, 48% GC, Tm: 66 °C

miR-135
miR-135 forward: 5′-CGATATGGCTTTTTATTCCTA -3′; 21 nt, 33% GC, Tm: 56 °C
miR-135 reverse: 5′-GAGCAGGGTCCGAGGT -3′; 16 nt, 69% GC, Tm: 67 °C

miR-140
miR-140 forward: 5′-GGGCAGTGGTTTTACCCTA -3′; 19 nt, 53% GC, Tm: 64 °C
miR-140 reverse: 5′-CAGTGCGTGTCGTGGAGT -3′; 18 nt, 61% GC, Tm: 68 °C

miR-143
miR-143 forward: 5′-AGTGCGTGTCGTGGAGTC-3′; 18 nt, 61% GC, Tm: 68 °C
miR-143 reverse: 5′-GCCTGAGATGAAGCACTGT-3′; 19 nt, 53% GC, Tm: 65 °C

miR-145
miR-145 forward: 5′-AGAGAACTCCAGCTG-3′; 15 nt, 53% GC, Tm: 56 °C
miR-145 reverse: 5′-GGCAACTGTGGGGTG-3′; 15 nt, 67% GC, Tm: 64 °C

miR-152
miR-152 forward: 5′-GGTTCAAGACAGTACGTGACT-3′; 21 nt, 48% GC, Tm: 64 °C
miR-152 reverse: 5′-CCAAGTTCTGTATGCACTGA-3′; 20 nt, 45% GC, Tm: 62 °C

miR-155
miR-155 forward: 5′-TTAATGCTAATTGTGATAGGGGT-3′; 23 nt, 35% GC, Tm: 61 °C
miR-155 reverse: 5′-CCTATCACAATTAGCATTAATT-3′; 22 nt, 27% GC, Tm: 55 °C

miR-210
miR-210 forward: 5′-CATAGATAGCCACTGCCCACA-3′; 21 nt, 52% GC, Tm: 67 °C
miR-210 reverse: 5′-GTGCAGGGTCCGAGGTATTC-3′; 20 nt, 60% GC, Tm: 68 °C

miR-218
miR-218 forward: 5′-TCGGGCTTGTGCTTGATC T-3′; 18 nt, 56% GC, Tm: 65 °C
miR-218 reverse: 5′-GTGCAGGGTCCGAGTG-3′’; 16 nt, 69% GC, Tm: 66 °C

miR-221
miR-221 forward: 5′-CCCAGCATTTCTGACTGTTG-3′; 20 nt, 50% GC, Tm: 64 °C
miR-221 reverse: 5′-TGTGAGACCATTTGGGTGAA-3′; 20 nt, 45% GC, Tm: 64 °C

miR-222
miR-222 forward: 5′-CGCAGCTACATCTGGCTACTG-3′; 21 nt, 57% GC, Tm: 68 °C
miR-222 reverse: 5′-GTGCAGGGTCCGAGGT-3′; 16 nt, 69% GC, Tm: 68 °C

miR-224
miR-224 forward: 5′-GCGAGGTCAAGTCACTAGTGGT-3′; 22 nt, 55% GC, Tm: 69 °C
miR-224 reverse: 5′-CGAGAAGCTTGCATCACCAGAGAA CG-3′; 26 nt, 54% GC, Tm: 72 °C

miR-320
miR-320 forward: 5′-AACGGAGAGTTGGGTCGAAA-3′; 20 nt, 50% GC, Tm: 66 °C
miR-320 reverse: 5′-TTGCCTCTCAACCCAGCTTT-3′; 20 nt, 50% GC, Tm: 67 °C

miR-365
miR-365 forward: 5′-ATAGGATCCTGAGGTCCCTTTCGTG-3′; 25 nt, 52% GC, Tm: 70 °C
miR-365 reverse: 5′-GCGAAGCTTAAAAACAGCGGAAGAGTTTGG-3′; 30 nt, 47% GC, Tm: 72 °C

miR-375
miR-375 forward: 5′-GGCTCTAGAGGGGACGAAGC-3′; 20 nt, 65% GC, Tm: 70 °C
miR-375 reverse: 5′-GGCAAGCTTTTTCCACACCTCAGCCTTG-3′; 28 nt, 54% GC, Tm: 74 °C

miR-424
miR-424 forward: 5′-AGGACGAAACACCCCCTATTCCTTGC-3′; 26 nt, 54% GC, Tm: 73 °C
miR-424 reverse: 5′-TAATGGATCCGAATACCTGCTCCTGA-3′; 26 nt, 46% GC, Tm: 69 °C

miR-494
miR-494 forward: 5′-GAAGATCTACGTCTGGTCTACCCAGTGC-3′; 28 nt, 54% GC, Tm: 72 °C
miR-494 reverse: 5′-GGGGTACCACCGAGAGTGGAGCCGGCAA-3′; 28 nt, 68% GC, Tm: 82 °C

miR-654
miR-654 forward: 5′-GGGATGTCTGCTGACCA-3′; 17 nt, 59% GC, Tm: 64 °C
miR-654 reverse: 5′-CAGTGCGTGTCGTGGA-3′; 16 nt, 63% GC, Tm: 65 °C

miR-720
miR-720 forward: 5′-GCGTGCTCTCGCTGGGG-3′; 17 nt, 76% GC, Tm: 73 °C
miR-720 reverse: 5′-GTGCAGGGTCCGAGGT-3′; 16 nt, 69% GC, Tm: 68 °C

miR-1246
miR-1246 forward: 5′-TGAAGTAGGACTGGGCAGAGA-3′; 21 nt, 52% GC, Tm: 67 °C
miR-1246 reverse: 5′-TTTGGGTCAGGTGTCCACTC-3′; 20 nt, 55% GC, Tm: 67 °C

Downstream miR-27 targets:

Riboflavin kinase (RFK)

RFK forward: 5′-CACCTGCCTTACTTCTGCCG-3′; 20 nt, 60% GC, Tm: 69 °C

RFK reverse: 5′-CCAACACTGGCCCAACCATAG-3′; 21 nt, 57% GC, Tm: 69 °C

Latent Transforming Growth Factor Beta Binding Protein 1 (LTBP1)

LTBP1 forward: 5′-CTGACGGCCACGAACTTCC-3′; 19 nt, 63% GC, Tm: 69 °C

LTBP1 reverse: 5′-GCACTGACATTTGTCCCTTGA-3′; 21 nt, 48% GC, Tm: 65 °C

INO80 complex subunit D (INO80D)

INO80D forward: 5′-ATAAGCCCTTGTGCTCATATAGC-3′; 23 nt, 43% GC, Tm: 64 °C

INO80D reverse: 5′-AGCGTTGGCTGTTATACTTGG-3′; 21 nt, 48% GC, Tm: 65 °C

Basic transcription factor 3 (BTF3)

BTF3 forward: 5′-CCAAGGAACAGTGATCCACTTT-3′; 22 nt, 45% GC, Tm: 65 °C

BTF3 reverse: 5′-AGCTGCTTTGTCTCAGCATGG-3′; 21 nt, 52% GC, Tm: 68 °C

Lysosomal thiol reductase (IFI30)

IFI30 forward: 5′-CCCCTCTGCAAGCGTTAGAC-3′; 20 nt, 60% GC, Tm: 68 °C

IFI30 reverse: 5′-CCCGCAGGTATAGATTGCCT-3′; 20 nt, 55% GC, Tm: 67 °C

Homologous to the E6-AP Carboxyl Terminus (HECT) ubiquitin protein ligase 2 (HECW2)

HECW2 forward: 5′-AAATCCCCAGATGCGGTACAC-3′; 21 nt, 52% GC, Tm: 67 °C

HECW2 reverse: 5′-CGGCTCTCAGAAGTCACCA-3′; 19 nt, 58% GC, Tm: 67 °C

Interleukin 2 (IL2)

IL2 forward: 5′-TACAAGAACCCGAAACTGACTCG-3′; 23 nt, 48% GC, Tm: 66 °C

IL2 reverse: 5′-ACATGAAGGTAGTCTCACTGCC-3′; 22 nt, 50% GC, Tm: 66 °C

Adenylate cyclase 1 (ADCY1)

ADCY1 forward: 5′-AGGCACGACAATGTGAGCATC-3′; 21 nt, 52% GC, Tm: 68 °C

ADCY1 reverse: 5′-TTCATCGAACTTGCCGAAGAG-3′; 21 nt, 48% GC, Tm: 65 °C

Eukaryotic translation initiation factor 5 (EIF5)

EIF5 forward: 5′- AGCGTGTCAGACCAGTTCTAT-3′; 21 nt, 48% GC, Tm: 65 °C

EIF5 reverse: 5′-CTGTCTTGATTCCATTGCCTTTG-3′; 23 nt, 43% GC, Tm: 64 °C

Nucleophosmin 1 (NPM1)

NPM1 forward: 5′-GGAGGTGGTAGCAAGGTTCC-3′; 20 nt, 60% GC, Tm: 68 °C

NPM1 reverse: 5′-TTCACTGGCGCTTTTTCTTCA-3′; 21 nt, 43% GC, Tm: 65 °C

Anthrax Toxin Receptor (ANTXR) cell adhesion molecule 1

ANTXR1 forward: 5′-ACAGTTGGCTCACAAATTCATCA-3′; 23 nt, 39% GC, Tm: 65 °C

ANTXR1 reverse: 5′-TCACTGGCCCTTTCAAATCCT-3′; 21 nt, 48% GC, Tm: 66 °C

Solute carrier family 23 member 2 (SLC23A2)

SLC23A2 forward: 5′-CTTCACTCTTCCGGTGGTGAT-3′; 21 nt, 52% GC, Tm: 67 °C

SLC23A2 reverse: 5′-TTTCCGTAGTGTAGATCGCCA-3′; 21 nt, 48% GC, Tm: 65 °C

Downstream miR-375 targets:

Rearranged L-myc fusion (RLF)

RLF forward: 5′-GTCATCGCCCCGTATCTCC-3′; 19 nt, 63% GC, Tm: 68 °C

RLF reverse: 5′-TGGCAAGTCGATATACCTCCA-3′; 21 nt, 48% GC, Tm: 65 °C

POC1 centriolar protein B (POC1)

POC1 forward: 5′-TTGTAACCAGCGTGCAGTTTT-3′; 21 nt, 43% GC, Tm: 65 °C

POC1 reverse: 5′-CAGAGTCTCACGGTTCTGTCT-3′; 21 nt, 52% GC, Tm: 66 °C

Embryonic lethal abnormal vision (ELAV)-like RNA binding protein 4

ELAVL4 forward: 5′-AACCTCTATGTTAGCGGCCTT-3′; 21 nt, 48% GC, Tm: 66 °C

ELAVL4 reverse: 5′-TGGACACTCCTGTGACTTGAT-3′; 21 nt, 48% GC, Tm: 65 °C

Colorectal cancer associated 2 (COLCA2)

COLCA2 forward: 5′-CCGGAGCCTTTGCTCAATTC-3′; 20 nt, 55% GC, Tm: 67 °C

COLA2 reverse: 5′-ACTGGCGAGTAACTGTAGTT-3′; 20 nt, 45% GC, Tm: 63 °C

Sperm associated antigen 9 (SPAG9)

SPAG9 forward: 5′-CAAGGCGGATCTAAAGCTACC-3′; 21 nt, 52% GC, Tm: 65 °C

SPAG9 reverse: 5′-TTGGCGCATCTGTAACCTTCA-3′; 21 nt, 48% GC, Tm: 67 °C

Centromere protein M (CENPM)

CENPM forward: 5′-GCGGACTCGATGCTCAAAGA-3′; 20 nt, 55% GC, Tm: 67 °C

CENPM reverse: 5′-TTCTGGAGACTGTATTTGCTGTG-3′; 23 nt, 43% GC, Tm: 64 °C

Recombination signal binding protein for immunoglobulin kappa J region (RBPJ)

RBPJ forward: 5′-CGGCCTCCACCTAAACGAC-3′; 19 nt, 63% GC, Tm: 68 °C

RBPJ reverse: 5′-TCCATCCACTGCCCATAAGAT-3′; 21 nt, 48% GC, Tm: 66 °C

### 4.6. Statistical Analysis

Absorbance readings from the NanoDrop 2000 Spectrophotometer and the ELx808 BioTek plate reader were exported into Microsoft Excel (Redmond, WA, USA). Descriptive statistics, including averages, were compiled and differences between experimental and control treatments were analyzed using two-tailed Student’s t-tests. Due to the potential for error using multiple two-way t-tests, verification of results was performed using Analysis of Variance (ANOVA). Significance levels were set at alpha = 0.05. In addition, Pearson’s correlation between biomarker expression (miRNA or miRNA target) as determined by average cycle threshold (CT) count and average chemotherapy resistance were calculated.

## 5. Conclusions

These data have demonstrated that chemotherapeutic resistance among commercially available oral cancer cell lines may be associated with differential regulation and expression of at least two miRNAs, miR-375 and miR-27. Moreover, this expression may influence specific downstream targets such as Rearranged L-myc fusion (RLF) and Nucleophosmin 1 (NPM1) expression—critical mediators of cancer growth and chemotherapy resistance. This provides pathways and functional mechanisms that can be explored in future studies of potential therapeutic interventions, as well as providing more immediate benefits in the identification of specific biomarkers that may identify tumor resistance and inform therapeutic decisions—a goal of personalized medicine and individualized therapy.

## Figures and Tables

**Table 1:** Analysis of RNA concentration and purity.

Cell Line	RNA Concentration [ng/uL]	RNA Purity A260:A280 Ratio	Secondary Purity A260:A230
SCC4	515.3 +/− 29.2	1.81	2.02
SCC9	488.1 +/− 35.1	1.74	2.06
SCC15	492.3 +/− 43.2	1.71	2.11
SCC25	501.2 +/− 55.4	1.76	2.08
CAL27	511.2 +/− 51.2	1.83	2.14
Average	501.6 +/− 42.8	1.77	2.08
Range	488.1–511.2	1.71–1.83	2.02–2.14

**Table 2:** Analysis of cDNA concentration and purity.

Cell Line	cDNA Concentration [ng/uL]	DNA Purity A260:A180 Ratio
SCC4	1566.2 +/− 101.3	1.81
SCC9	1455.2 +/− 94.1	1.84
SCC15	1501.3 +/− 99.8	1.84
SCC25	1438.4 +/− 83.3	1.88
CAL27	1522.1 +/− 98.2	1.82
Average	1496.6 +/− 95.3	1.84
Range	1438.4–1566.2	1.81–1.88

**Table 3:** Association between miRNA expression and potential downstream targets in oral cancer cell lines.

	SCC9 Resistance Range: −3.3% to −18.6% Average: −12.3%	SCC25 Resistance Range: −3.6% to −11.9% Average: −8.8%	SCC4 Resistance Range: −32.5% to −37.9% Average −35.2%	CAL27 Resistance Range: −40.1% to −44.3% Average −41.7%	SCC15 Resistance Range: −62.7% to −68.3% Average −65.5%
miR-27	n.d.	n.d.	miR-27 CT value: 20	miR-27 CT value: 20	miR-27 CT value: 15
miR-27 target	NPM1 CT value: 10	NPM1 CT value: 10	n.d.	n.d.	n.d.
miR-375	miR-375 CT value: 25	miR-375 CT value: 30	n.d.	n.d.	n.d.
miR-375 target	RLF CT value: 20	RLF CT value: 15	n.d.	n.d.	n.d.
miR-375 target	n.d.	n.d.	POC1 CT value: 35	POC1 CT value: 35	POC1 CT value: 35

## Data Availability

The data presented in this study are available on request from the corresponding author.
